# Evaluation of Post Cessation Weight Gain in a 1-Year Randomized Smoking Cessation Trial of Electronic Cigarettes

**DOI:** 10.1038/srep18763

**Published:** 2016-01-05

**Authors:** Cristina Russo, Fabio Cibella, Pasquale Caponnetto, Davide Campagna, Marilena Maglia, Evelise Frazzetto, Enrico Mondati, Massimo Caruso, Riccardo Polosa

**Affiliations:** 1 Centro per la Prevenzione e Cura del Tabagismo, Azienda Ospedaliero-Universitaria “Policlinico-V. Emanuele”, Università di Catania, Catania, Italy; 2 Institute of Internal Medicine, S. Marta Hospital, Azienda Ospedaliero-Universitaria “Policlinico-V. Emanuele”, Università di Catania, Catania, Italy; 3 National Research Council of Italy, Institute of Biomedicine and Molecular Immunology, Palermo, Italy

## Abstract

Stop smoking it is often associated to weight gain that is one of the most important causes for relapse. This is the first study to describe long-term changes in body weight in smokers invited to quit or reduce smoking by switching to ECs. Conventional cigarettes consumption and body weight were measured prospectively in a randomized controlled trial of smokers invited to switch to ECs. Post cessation weight changes from baseline at week-12, -24 and -52 were compared among 1) high, medium and zero nicotine strength products and 2) pooled continuous smoking failure, smoking reduction and abstinence phenotypes. Saliva cotinine levels and appetite levels were also measured. No significant changes in body weight were observed among high, medium and zero nicotine strength products. Differences among continuous smoking phenotypes were significant only at week-12 (p = 0.010) and week-24 (p = 0.012) with quitters gaining 2.4{plus minus}4.3 Kg and 2.9{plus minus}4.4 Kg respectively. However, weight gain at week-52 (1.5{plus minus}5.0 Kg) was no longer significant compared to Failures and Reducers. No confounding factors could explain the significant changes in body weight. Smokers who quit smoking by switching to ECs may limit their post-cessation weight gain, with substantial reversal in weight gain being manifest at late time points.

Cigarette smoking is the most important cause of avoidable premature mortality in the world and quitting is known to reduce risk of fatal diseases such as lung cancer, acute coronary artery disease, strokes, end-stage chronic obstructive pulmonary disease and other cancers^1^. The World Health Organization (WHO) Framework Convention on Tobacco Control (FCTC) advises that one of the key actions to reduce health burden associated with combustible tobacco use is to encourage abstinence among smokers^2^. Current (i.e. nicotine replacement therapy-NRT, bupropion and varenicline) and emerging smoking cessation drugs are known to increase the likelihood of quitting smoking, particularly if combined with counseling programs^3^,^4^.

However, while stop smoking results in considerable health improvements, it is often accompanied by weight gain. The link between smoking and body weight is well established. Cross sectional studies show that, on average, smokers weigh less than non-smokers, and former smokers weigh more than both smokers and non-smokers^5^. A large prospective study has shown that adolescents who initiate smoking gain less weight than their non-smoking peers^6^. Cohort studies also show that people who stop smoking gain weight^5^,^7^,^8^,^9^. Nicotine (in tobacco cigarettes) is known to suppress appetite and to increase resting metabolic rate^7^. Therefore, weight gain in those who quit smoking is probably due to the combination of a decline in resting energy expenditure at a time when appetite is increased.Of note, weight increase after quitting is one of the most important causes for relapse into tobacco smoking, in particular for wome^10^,^11^

Several strategies have been tested for weight-concerned smokers wanting to stop smoking. A Cochrane review that examined the effectiveness of first line antismoking medications (i.e. NRT, bupropion, and varenicline) on limiting post cessation weight gain^12^ showed only modest results, with NRT, bupropion, and varenicline reducing weight gain only by 0.5 kg, 1.1 kg, and 0.4 kg, respectively. Moreover, this advantage was soon lost after treatment discontinuation.Although regular diet and physical activity might be effective to prevent post cessation weight gain, recent metaanalyses have shown that dietary strategies or exercise programs have been inconsistent at controlling post cessation weight gain^12^,^13^.Consequently, the need for novel and more efficient approaches is unquestionable.

The nicotinic and non-nicotinic effects of electronic cigarettes (ECs) might be exploited for weight-concerned smokers wanting to stop smoking. Electronic cigarettes (ECs) are battery-operated devices designed to vaporise nicotine without burning tobacco. These consumer products share many similarities with smoking in the behavioural aspect of their use^14^,^15^. Users are predominantly smokers, who report using them long-term as an alternative for conventional cigarettes, to reduce cigarette consumption or quit smoking, to relieve tobacco withdrawal symptoms, and to continue having a ‘smoking’ experience^16^,^17^, but with much reduced health risks^18^. Data from a recent prospective randomized controlled trial have shown that ECs can aid smoking cessation and reduction with long-term quit rates of up to 8.7% in smokers not intending to quit^19^. Moreover, a recent meta-analysis including 1,242 smokers with complete data on smoking cessation after switching to ECs for a minimum period of six months reported a 18% quit rate^20^.

To the best of our knowledge, post cessation weight gain after switching to ECs use has never been investigated. Herein we have used body weight measured at baseline and at 12-, 24- and 52-week study visits from the ECLAT (EffiCiency and safety of an eLectronic cigAreTte)study^19^-a prospective 12-month double-blind, controlled, randomized clinical three-arm trial designed to evaluate smoking reduction, smoking abstinence and adverse events in “healthy” smokers not intending to quit after switching to a popular EC brand (‘Categoria’; Arbi Group Srl, Italy). Body weight changes from baseline were compared amongst 1) high, medium, and zero nicotine strength products and 2) pooled continuous smoking failure, smoking reduction and abstinence phenotypes.

Our working hypothesis is that substitution of conventional cigarettes with ECs might limit post-cessation weight gain becausethese products- by mimicking the physical, visual, sensorial, and behavioural experience of conventional smoking- can replace the oral satisfaction without which many abstainers would fill their sense of emptiness and hunger with compulsiveeating.

## Methods

Details of participants’ characteristics and study design have been previously described^19^. The ERB of the “Policlinico-Vittorio Emanuele” Hospitals approved the study and participants gave written informed consent prior to participation in the study. The methods were carried out in accordance with the approved guidelines.

### Participants

Regular smokers from Catania (Italy) not intending to quit were invited to try ECs (“Categoria”, Arbi Group Srl, Italy) as a less harmful alternative to tobacco smoke that could be freely used as a complete substitute for conventional cigarettesfrom June 2010 to February 2011.

Subjects were made aware that the purpose of the current assessments was to quantify reductions in cigarette consumption by switching to EC use and the impact on their body weight on a regular basis at follow-up visits. No financial incentive was offered for participation.

Inclusion criteria were: (a) smoke ≥10 tobacco cigarettes per day (cig/day), for at least the past five years, (b) age 18–70 years, (c) in good general health; (d) not currently attempting to quit smoking or wishing to do so in the next 30 days(this was verified at screening by the answer “NO” to both questions “Do you intend to quit in the next 30 days?” and “Are you interested in taking part in one of our smoking cessation programs?”), and (e) committed to follow the trial procedures.

Exclusion criteria were: (a) history of symptomatic cardiovascular disease, symptomatic respiratory disease, psychiatric disorder or major depression; (b) regular medication use; (c) current or past history of alcohol abuse; (d) use of smokeless tobacco or nicotine replacement therapy, and (e) pregnancy or breastfeeding.

### Study Design

**Figure 1:**
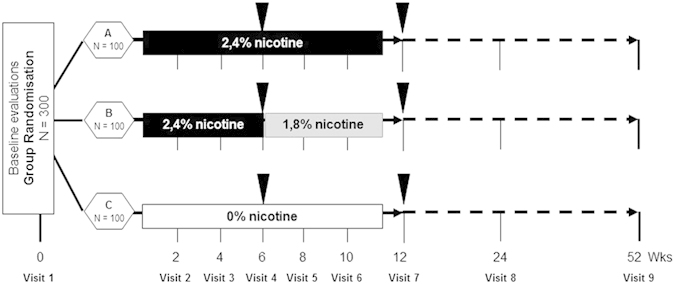
Schematic diagram of the ECLAT study design. Smokers not currently attempting to quit smoking or wishing to do so in the next 30 days were randomized in three study groups: group A (receiving 12 weeks of “Original” 2.4% nicotine cartridges), group B (receiving 6-weeks of “Original” 2.4% nicotine cartridges and a further 6 weeks with “Categoria” 1.8% nicotine cartridges), and group C (receiving 12 weeks of “Original” 0% nicotine cartridges). Participants in each group were prospectively reviewed for up to 52-weeks during which smoking habits, eCO levels, and MNWS were assessed at each study visits. Body weight was measured at baseline, and at week-12, week-24 and week-52. Saliva samples were collected at week-6 and at week-12 (closed triangles) for cotinine measurement in those who stated they had not smoked and with an eCO ≤7 ppm.

Eligible participants were enrolled into a prospective 12-month randomized, controlled trial consisting of nine office visits at the University Hospital’s smoking cessation clinic (Centro per la Prevenzione e Cura del Tabagismo-CPCT; Universita‘ di Catania, Italy) to assess biochemically verified (by exhaled carbon monoxide-eCO monitoring) cigarette consumption. Body weight was measured at baseline and at week-12, week-24 and week-52. Participants were randomized into three study arms to receive EC kits with either“Original 2.4%” cartridges for 12 weeks(Group A), or “Original 2.4%” for 6 weeks and a further 6 weeks with“Categoria 1.8%”(Group B), or“Original 0%” cartridges for 12 weeks(Group C) (Fig. 1). The randomization sequence was generated using a computer atthe Hospital pharmacy and blinding was ensured by the identical appearance of the cartridges.

At baseline, socio-demographic factors, smoking history, Fagerstrom Test for Cigarette Dependence (FTCD) scores, levels of eCO (Micro CO, Micro Medical Ltd, UK) and withdrawal symptoms by Minnesota Nicotine Withdrawal Scale (MNWS) were annotated. The MNWS measures craving, irritability, anxiety, difficulty concentrating, restlessness, depression, insomnia, and increased appetite. The item “increased appetite” is scored on a scale ranging from 0 (not at all present) to 4 (extreme). Body weight and height were also recorded. Participants were then given a free EC kit with a full supply of cartridges, and were trained on how to correctly use the product. They were told to use the study product ad libitum (but up to a maximum of 4 cartridges/day) in the anticipation of reducing cigarette smoking, and to take notes of the daily consumption of conventional cigarettes and cartridge use in their study diaries.

Participants were then invited to return to the CPCT at follow-up visits a) to receive further free supply of cartridges and study diaries for the residual study periods, b) to record their eCO levels, c) to have their body weight measured (at week-12, week-24 and week-52 only), d) to measure MNWS, and e) to return completed study diaries and unused study products.

Saliva samples were collected at week-6 (study visit 4) and at week-12 (study visit 7) for cotinine measurement in those who stated they had not smoked (not even a puff) and with an eCO ≤7 ppm. Participants were asked to chew a small cotton roll (TR0N00RU2, Dentalica, Milano, Italy) for 60 seconds. Cotton rolls were placed into polypropylene tubes and stored at −20 °C until use. Saliva samples were analysed in duplicate for cotinine analysis by gas chromatography^21^.

By week-12 study visit, no more cartridges were provided, but participants were advised to continue using their EC if they wish to do so.

### Body weight measurements

At each visit, study participants removed shoes and heavy clothing and were weighed using a mechanical column scale (Seca, Intermed Srl, San Giuliano Milanese, Italia). Height measurements were taken at the baseline visit by using a standing scale slide bar.

### Products Tested

The “Categoria” EC (model “401”) was used in this study. It is a three-piece model that closely resembles a conventional cigarette, activated by a rechargeable 3.7 V-90 mAh lithium-ion battery. Disposable cartridges used in this study were of three different types, but of identical appearance: “Original 2.4%” (2.27 ± 0.13% nicotine), “Categoria 1.8%” (1.71 ± 0.09% nicotine) and “Original 0%” without nicotine (“sweet tobacco” aroma). Detailed toxicology and nicotine content analyses of these cartridges had been carried in a laboratory certified by the Italian Institute of Health and can be found at: http://www.liaf-onlus.org/public/allegati/categoria1b.pdf

The “Categoria” EC kit and cartridges were provided free of charge by the local distributor, Arbi Group Srl, Italy.

### Smoking Phenotypes and Study Outcomes

Smoking abstinence was defined as complete self-reported abstinence from tobacco smoking (not even a puff) since the previous study visit, which was biochemically verified by eCO levels of ≤7 ppm. Smokers in this category are classified as Quitters.

Smoking reduction was defined as sustained self-reported ≥50% reduction in the number of cig/day from baseline (eCO levels were measured to verify smoking status and confirm a reduction compared to baseline). Smokers in this category are classified as Reducers.

Smokers who were not categorised in the above categories were classified as Failures.

The primary outcome of interest was the % change in body weight from baseline to the final follow-up visit at week-52.

### Statistical Analyses

To investigatethe effect of different nicotine concentration on weight changes, body weight values were compared among the study arms (Group A–C: per-protocol analysis). Descriptive data are presented as means ± standard deviations (SD) or medians and interquartile range (IQ) for normally and not normally distributed variables respectively. Baseline differences among groups (A–C) were evaluated by χ^2^ test for categorical variables, and by one-way analysis of variance (ANOVA) and Fisher protected LSD for continuous parametric variables. Difference and correlation between non-parametric variables were investigated by means of Kruskall-Wallis test, Mann-Whitney U-test, and Spearman Rank Correlation test. Within and between subject changes in time trends of body weight were evaluated by repeated measures ANOVA.

To investigate the effect of smoking phenotype on weight changes, these were compared among continuous smoking phenotypes (Quitters, Reducers and Failures), irrespective of the study arm that each participant was assigned to. Among these subjects, a repeated measures ANOVA model was built: body weight values at different time points were entered into the model as within factor, while continuous smoking phenotype was entered as between factor. Moreover, weight changes (with respect to baseline) at wk-12, -24, and -52 were introduced into multiple linear regression models (as continuous dependent variables) and tested against phenotype classification, and some confounders/effect modifiers including: age, gender, high FTND at baseline (≥7), no. of cigarettes smoked at baseline, BMI at baseline, and changes in hunger levels as derived from the MNWS (a MNWS-increased appetite score of 0 was categorized as normal appetite, whereas scores ≥ 1 was categorized as increased appetite).

The analyses were carried out using Statistical Package for Social SciencesIBM SPSS Statistics (IBM, Armonk, New YorkSPSS Inc., Chicago, IL) for Windows version 20.0 and p values of 0.05 was considered significant.

## Results

After screening, a total of 300 [M 190, F 110; mean (±SD) age of 44.0 (±12.5) years] regular smokers (median [IQ range] pack/yrs of 24.9 [14.0-37.0]) were eligible and consented to participate in the study (Table 1). Baseline characteristics were similar among study groups A, B, and C, with the exception of participants’ age in group A vs group C (45.9 ± 12.8 vs 42.2 ± 12.5; p = 0.04, Fisher’s least significant difference). No difference was observed in body weight.

Two-hundred-twenty-five subjects (75.0%) returned at week-12, 211 (70.3%) at week-24, and 183 (61.0%) for their final follow-up visit at week-52. Baseline characteristics of those who were lost to follow-up were not significantly different from participants who completed the study (with the exception of gender) and no significant difference was evident in drop-out rates among study groups at any study visit. Overall, reduction and quit rates (%) in the ECLAT study were not significantly different among study groups. In particular, at week-52 quitters were 13% in Group A, 9% in Group B, and 4% in Group C. More details about success rates with ECs have been reported in the ECLAT study^19^.

Among 183 subjects completing their final follow-up visit at week-52, 145 could be categorized as continuous smoking phenotype (Quitters, Reducers and Failures). Their baseline characteristics are illustrated in Table 2. In these 145 subjects, body weight was 70.9 ± 15.6 Kg at baseline, 71.6 ± 15.9 Kg at week-12, 71.5 ± 13.0 Kg at week-24, and 71.3 ± 13.0 Kg at week-52.

**Figure 2:**
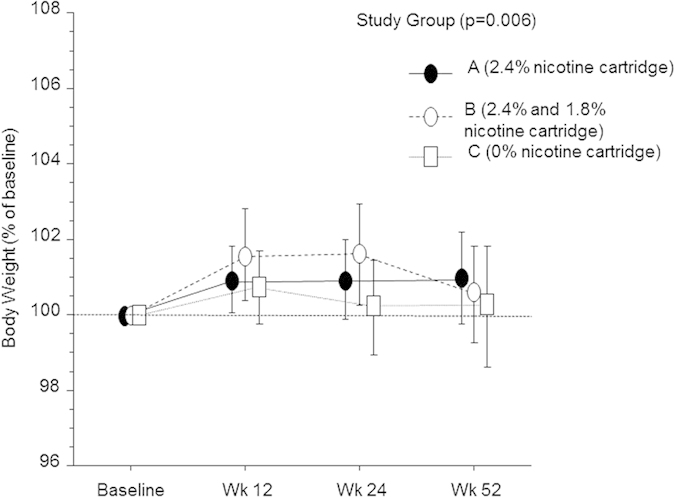
Time-course in body weight changes (expressed as percent of baseline ± 95% confidence intervals) at week-12, -24, and -52 in subjects with continuous smoking phenotype classification, separately for each study group (A–C). Repeated measures ANOVA demonstrated a significant within factor (body weight) difference (p = 0.006), but no significant effect among study groups for body weight.

In Fig. 2 the time trends of body weight changes (expressed as % of baseline) are presented for all 145 subjects, irrespective of smoking phenotype. Repeated measures ANOVA demonstrated a significant within factor (body weight) difference (p = 0.006), but no significant effect were observed among study groups for body weight (i.e., between factor). Because no significant difference was found among study groups, for the purposes of the present study, body weight data from all study groups were combined together for subsequent analyses.

**Figure 3:**
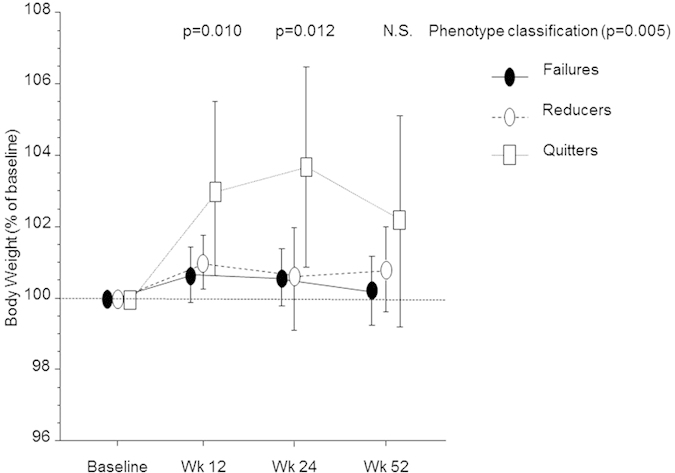
Time-course in body weight changes (expressed as percent of baseline ± 95% confidence intervals) at week-12, -24, and -52, separately for each continuous smoking phenotype classification (quitters, reducers, failures). Repeated measures ANOVA showed that within factor (body weight) differences were significant (p = 0.005); the effect of between phenotype classification showed a significant effect at week-12 (p = 0.010) and week-24 (p = 0.012), but not at week-52.

Repeated measures ANOVA also showed that the effect of phenotype classification (i.e., the between subject factor) was significant only at week-12 (p = 0.010) and week-24 (p = 0.012) with quitters gaining 2.4 ± 4.3 Kg and 2.9 ± 4.4 Kg at week-12 and week-24 respectively. However, weight gain at week-52 in quitters was reduced down to 1.5 ± 5.0 Kg and no longer significantcompared to Failures and Reducers (Fig. 3).Weight changes from baseline (kg ± SD) in quitters only (separately for groups A–C, at each time point) were never statistically significant.Although more weight gain (kg ± SD) was observed in quitters who stopped using EC compared to quitters who were still using EC (combined for groups A–C), this was notnever significant at any study time points.

**Figure 4:**
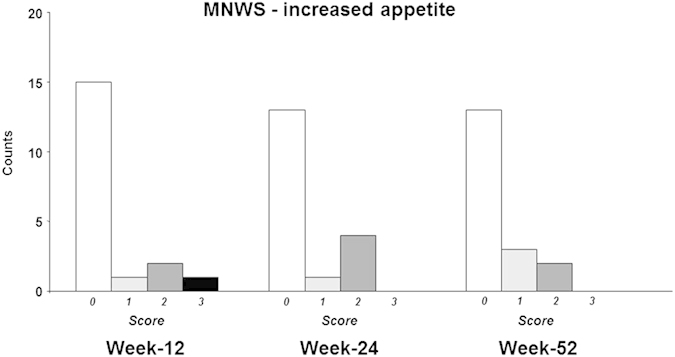
Frequency distribution of incremental MNWS-increased appetite scores for quitters at week-12, -24, and -52; score 0 = “not at all present” (white bars); score 1 = “slight” (light grey bars); score 2 = “moderate” (dark grey bars); score 3 = “quite a bit” (black bars). Please note that “extreme” increased appetite (i.e. score 4) was never reported.

Frequency distribution of incremental MNWS-increased appetite scores for quitters is shown in Fig. 4. Increased appetite (MNWS-increased appetite scores ≥ 1) was reported in 21.1%, 27.7% and 27.7% of quitters at week-12, -24, and -52, respectively. Similar frequency distribution was also observed at earlier time-points.

In multiple regression models we evaluated for weight changes (with respect to baseline) at wk-12, -24, and -52 as dependent variable, and age, gender, high FTND at baseline (≥7), no. of cigarettes smoked at baseline, and BMI at baseline, MNWS appetite score, and phenotype classification as independent variables.A MNWS-increased appetite score ≥ 1 showed a significant effect only at week-12 (p = 0.036).According to the results provided by repeated measures ANOVA, when corrected for the confounding factors mentioned earlier, multiple regression models showed thatfailure in reducing or quitting tobacco smoking has a protective effect on weight gain compared to successfully quitting at Week-12 and Week-24 (p <0.0001 and p = 0.004, respectively). In fact,at both time points, the beta coefficientsfor failures and reducers provided by the multiple regression models (and thus corrected for confounders)are negative and significant with respect to quitters (i.e., the reference group for this analysis). Interestingly, this effect disappears at Week-52, when the previously significant effect of smoking phenotype on weight gain is no longer present. (Table 3).

**Figure 5:**
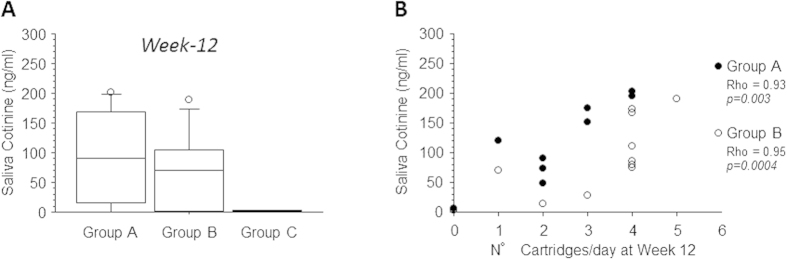
(**A**)-Box plots representation of saliva cotinine levels in those who stated they did not smoke and with an eCO ≤7 ppm at week-12, separately for each study group (**A–C**). The difference between groups A and B was assessed by Mann-Whitney U test and found not to be significant. Bars indicate (from the bottom to the top) 10 th, 25 th, 50 th (median), 75 th, and 90 th percentiles. Values below 10 th and above 90 th percentiles (outliers) are shown as circles. (**B**)-Relationships between saliva cotinine levels and number of e-cigarettes cartridges/day at week-12, separately for study groups A and B. The strength of the association was assessed using Spearman Rank Correlation test and found to be highly significant in both study groups.

In those who stated they did not smoke (not even a puff) and with an eCO ≤7 ppm, saliva cotinine levels by the end of the intervention phase (i.e. week-12) were not significantly different between group A and B (Fig. 5A); their median (IQR) concentration being 91.0 ng/ml (16.3–169.4) in Group A and 69.8 (0.9–104.9) ng/ml in Group B at week-12. Correlations between saliva cotinine levels and number of cartridges/day were highly significant for study groups A and B (Rho = 0.93 for group A, p = 0.003; Rho = 0.95 for group B, p = 0.0004). (Fig. 5B). As expected, saliva cotinine levels in the no-nicotine group (group C) were generally close to limit of detection (Fig. 5A).

## Discussion

While stopping smoking results in considerable health improvements, it is accompanied by weight gain in four out of five quitters^22^. The problem of post-cessation weight gain can have important health consequences considering that smoking and obesity are both risk factors for cardiovascular disease, and some cancers^23^. Furthermore, smoking and obesity are also risk factors for type II diabetes^24^,^25^.

Consequently, the need for novel and more efficient approaches is unquestionable.

This is the first study to evaluate post cessation weight gain in smokers who reduced or quit smoking by using ECs in a randomized control trial. Details of quit and reduction (i.e. ≥50% smoking reduction from baseline) rates for this study population have been reported previously^19^.

Weight gain is observed in more than 80% of smokers who are successful in quitting smoking^22^. In a meta-analysis of 62 prospective studies recording weight changes in abstinent smokers, the average weight gain at 3, 6, and 12 months after quitting was 2.9, 4.2 and 4.7 kg, respectively^26^. In our study, quitters gained on average 2.4 Kg and 2.9 Kg at Week-12 and Week-24, respectively. Thus, the weight gain we measured after switching was relatively small. This could be due to the small number of women in the study and/or to low incidence of reported increase in appetite. Bearing in mind that women tend to gain more weight than men after smoking cessation^27^, if the proportion of women who quit in a given study is substantially different from men, the level of post-cessation weight gain will probably reflect the relative contribution of the female population to the overall study sample. Female participation in the ECLAT study was 40.7%; most importantly, amongst the 18 continuous quitters only 4 were women (see Table 2). Increased appetite was described only in two to three out of ten quitters with a significant effect for post-cessation weight gain only at week-12. It is possible that by providing a coping mechanism for conditioned smoking cues EC use could mitigate hunger associated with smoking abstinence. Moreover, EC use appears to improve cognitive effects during tobacco abstinence^28^. Taken together these mechanisms suggest that EC use may limit post-cessation weight gain.

Based on the notion that nicotine is a well-known appetite suppressants and increases resting metabolic rate^7^, we expected to see more substantial control of post-cessation weight gain with substitution of conventional cigarettes with ECs. However, it must be noted that saliva cotinine levels in the EC users were well below the concentrations range normally found in regular smokers^29^ or experienced EC users^30^. This is not surprising considering that the model under investigation-equipped with a small 90 mAh lithium-ion battery that allows only about 50–70 puffs-is not very efficient at delivering nicotine^31^. Hence, it is likely that the significant (but small) post–cessation weight gain observed at Week–12 and Week–24 after switching is also consequence of the very low nicotine absorption with the product under investigation.

Although these low nicotine levels were unlikely to allow efficient appetite suppression, the observed weight gain was not consistently related to appetite increase.Moreover, none of the confounding factors included in the multiple regression analysis models (i.e. age, gender, high FTND at baseline, number of cig/die at baseline, and BMI at baseline-Table 3) showed a significant effect.

Despite the significant (but modest) post-cessation weight gain at Week-12 and Week-24, a significant increase in body weight from baseline was no longer observed in those who were abstinent at Week-52. This is unusual and probably specific to quitters of a smoking cessation RCT who have switched to ECs. Our data do not allow us to specify what biobehavioral processes underlying the complex dynamics of these changes in body weight and multiple factors and interactions must be considered. Nonetheless post-cessation weight gain reversal at Week-52 is a key informative finding of the present study and requires explanation.

Abundance of calorically dense food and being less active during the winter months and winter holyday season can contribute to significant weight gain^32^,^33^. Given that more than 70% of study participants were enrolled in autumn (from September to November 2010), it is possible that imbalance in the rate of recruitment could have contributed to post–cessation weight gain observed at week–12 and week–24 (i.e. well into the winter season). The subsequent physiologic reduction in weight gain at week–52 could be consequence to returning to a less calorically dense diet and being more active during the summer months.

Often smokers cope with high stress levels during quitting by substituting their cigarette oral gratification with food. By perpetuating their smoking rituals with EC use, the quitting process for our group of smokers not planning to quit was easy, spontaneous and painless. For these individuals, EC use might have played a role by boosting smokers confidence in their ability to abstain from smoking as well as to lose weight at later time points. Likewise, we can also speculate that the surprisingly modest weight gain at earlier time points might have motivated quitters to pay increasing attention to their weight and eating thus accounting for the observed reversal in post-cessation weight gain at week-52.

Our RCT has the advantage of an interventional prospective trial approach, which minimizes the possibility of reverse causality of case-control and cross-sectional studies. Smoking abstinence was biochemically verified at each study visit. The effects of specific continuous smoking phenotypes were investigated on body weight gain in the same smokers over several time points for up to one year. Moreover, post-cessation weight gain was tested against a number of confounding factors.

There are however some limitations. Firstly, participants in this study may represent a self-selected sample (e.g. smokers not intending to quit switching to ECs), which is not representative of all smokers quitting or reducing tobacco smoking, however forms a good cohort of participants to ascertain the effects on post-cessation weight gain. Secondly, sample size was small in some smoking phenotype subgroup cohorts due to high attrition rates (not uncommon in smoking cessation studies), lack of financial incentive to study participants, and use of a continuous smoking phenotype classification for the statistical analyses. Nonetheless, we obtained highly significant results in this study. Thirdly, findings are likely to be product specific and cannot be generalized to the hundreds of other ECs on the market. Additionally, confounding factors (e.g. dietary habits, recreational exercise), which may have had an influence on weight gain were not assessed. Last but not least, the findings reported from this smoking population of Sicilian residents may not be valid for other population samples.

## Conclusion

By substantially reducing tobacco consumption and minimizing post-cessation weight gain, EC-based interventions may improve smokers’ overall cardiovascular and metabolic risk profile^34^. It is important that research continues in this area because the negative effects of obesity could outweigh the health benefits achieved through reductions in smoking prevalence^35^,^36^. Although replication of our findings in larger prospective studies using more representative population samples and with a large assortment of ECs models will be required, the data presented here may still prove helpful to researchers, policy makers, regulators, healthcare providers and consumers in a context where virtually no information about ECs is available.

## Additional Information

**How to cite this article**: Russo, C. *et al.* Evaluation of Post Cessation Weight Gain in a 1-Year Randomized Smoking Cessation Trial of Electronic Cigarettes. *Sci. Rep.* **6**, 18763; doi: 10.1038/srep18763 (2016).

## Figures and Tables

**Table 1:** Baseline characteristics of study participants for the overall sample and separately for each treatment arms.

	Overall sample (No. = 300)	Group A 12 wks nicotine 2.4% (No. = 100)	Group B 6 wks nicotine 2.4% 6 wks nicotine 1.8% (No. = 100)	Group C No nicotine (No. = 100)	P value
Gender (Males/Females)	190/110	61/39	66/34	63/37	NS
Age (yrs, mean ± SD)	44.0 ± 12.5	45.9 ± 12.8	43.9 ± 12.2	42.2 ± 12.5	0.04*
Pack/yr (median [IQR])	24.9 (14.0–37.0)	24.0 (14.3–37.0)	25.3 (16.9–38.8)	25.5 (12.0–35.0)	NS
Cig/day (median [IQR])	20.0 (15.0–25.0)	19.0 (14.0–25.0)	21.0 (15.0–26.0)	22.0 (15.0–27.0)	NS
eCO (ppm, median [IQR])	20.0 (15.0–28.0)	19.0 (15.5–29.0)	22.0 (16.0–29.0)	19.5 (14.0–28.0)	NS
FTND (mean ± SD)	5.8 ± 2.2	5.6 ± 2.3	6.0 ± 2.1	5.8 ± 2.2	NS
Past attempts to quit (% yes)	51	56	48	47	NS
Body Weight (kg, mean ± SD)	75.0 ± 15.0	74.0 ± 14.2	76.1 ± 15.3	74.8 ± 15.7	NS
BMI (kg/m^2^, mean ± SD)	25.8 ± 4.6	25.7 ± 4.2	25.9 ± 4.5	25.9 ± 5.1	NS

Abbreviations: SD, standard deviation; IQR, interquartile range; pack/yrs, pack-years; cig/day, cigarettes smoked per day; eCO, exhaled carbon monoxide; FTND, Fagerstrom Test for Nicotine Dependence.

Differences among groups were evaluated by χ^2^ test for categorical variables, one-way analysis of variance (ANOVA) and Fisher protected LSD, and Kruskall-Wallis test for continuous and not normally distributed variables, respectively.

^*^between A and C groups (ANOVA, Fisher’s Least Significant Difference).

**Table 2:** Baseline characteristics of study participants with continuous smoking phenotypes classification (No. = 145), separately for classification at week-52.

	Failures (No. = 93)	Reducers (No. = 34)	Quitters (No. = 18)	P value
Gender (M/F)	50/43	22/12	14/4	0.13*
Age (yrs, mean±SD)	41.6 ± 13.0	45.4 ± 14.4	44.8 ± 10.5	0.28**
Pack/yr (median, IQ range)	24.5 (11.1–35.0)	28.3 (15.0–45.0)	23.0 (16.8–33.6)	0.30***
Cig/day (median, IQ range)	20 (15–25)	18 (15–30)	19 (15–20)	0.40***
eCO (ppm, median, IQ range)	21 (14–29)	20 (15–26)	17 (12–20)	0.11***
FTND (mean ± SD)	5.9 ± 2.1	5.2 ± 2.1	5.1 ± 2.3	0.18**
Weight (kg, mean ± SD)	70.7 ± 12.5	69.6 ± 12.4	74.4 ± 13.5	0.40**
BMI (kg/m^2^, mean ± SD)	26.7 ± 3.8	24.1 ± 3.8	25.7 ± 4.2	0.38**

^*^χ^2^ test.

^**^one-way analysis of variance (ANOVA) and Fisher protected LSD.

^***^Kruskall-Wallis test.

**Table 3:** Parameter estimated by multiple regression analysis models for weight changes at week-12, -24, and -52 as dependent variables and smoking phenotype (Failures and Reducers vs Quitters), gender (female vs male), FTND at baseline (low vs high), age, number of cig/die at baseline, BMI at baseline, and MNWS-increased appetite (score 0 vs scores ≥ 1) as independent variables.

	Weight changes at week 12				Weight changes at week 24				Weight changes at week 52			
			95% confidence interval				95% confidence interval				95% confidence interval	
Parameter	β coefficient	*p*	Lower	Upper	β coefficient	*p*	Lower	Upper	β coefficient	*p*	Lower	Upper
Failures*	−2.443	***0.000***	−3.745	−1.141	−2.570	***0.004***	−4.285	−0.856	−1.332	*0.142*	−3.115	0.452
Reducers*	−2.191	***0.002***	−3.567	−0.816	−2.881	***0.003***	−4.778	−0.983	−0.775	*0.445*	−2.775	1.225
Female**	0.801	*0.083*	−0.106	1.708	1.059	*0.052*	0.008	2.126	1.032	*0.091*	−0.168	2.231
FTND <7 at baseline***	−0.156	*0.761*	−1.168	0.855	0.241	*0.694*	−0.967	1.448	0.959	*0.163*	−0.393	2.311
Age (yrs)	−0.029	*0.084*	−0.062	0.004	−0.014	*0.484*	−0.053	0.025	−0.006	*0.778*	−0.050	0.038
No. of cig/die at baseline	0.035	*0.196*	−0.018	0.088	0.014	*0.669*	−0.050	0.078	−0.016	*0.666*	−0.087	0.056
BMI at baseline (kg/m^2^)	0.052	*0.364*	−0.061	0.166	0.038	*0.580*	−0.097	0.173	0.051	*0.509*	−0.100	0.202
MNWS-increased appetite#	−0.971	***0.036***	−1.879	−0.064	−0.466	*0.408*	−1.576	0.645	−0.046	*0.950*	−1.502	1.410

^*^Ref: Quitters.

^**^Ref: Male.

^***^Ref: FTND ≥7 at baseline.

^#^Ref: MNWS-increased appetite score >0 .

In the MNWS, participants were also asked to rate about increased appetite by marking a number from 0 to 4; 0 = being “not at all”; 1 = being “slight”; 2 = being “moderate”; 3 = being “quite a bit”; and 4 = being “extreme”). A MNWS-increased appetite score of 0 was categorized as normal appetite, whereas scores ≥ 1 was categorized as increased appetite.
